# Large-scale polymorphism of heterochromatic repeats in the DNA of *Arabidopsis thaliana*

**DOI:** 10.1186/1471-2229-7-44

**Published:** 2007-08-16

**Authors:** Jerry Davison, Anand Tyagi, Luca Comai

**Affiliations:** 1 University of Washington, Department of Biology, Box 355325, Seattle, WA 98195-5325, USA; 2 University of the South Pacific, School of Pure and Applied Sciences, Department of Biology, PO Box 1168, Suva, Fiji; 3 University of California at Davis, Section of Plant Biology and the UC Davis Genome Center,451 E. Health Sciences Drive, Davis, CA 95616, USA

## Abstract

**Background:**

The composition of the individual eukaryote's genome and its variation within a species remain poorly defined. Even for a sequenced genome such as that of the model plant *Arabidopsis thaliana *accession Col-0, the large arrays of heterochromatic repeats are incompletely sequenced, with gaps of uncertain size persisting in them.

**Results:**

Using geographically separate populations of *A. thaliana*, we assayed variation in the heterochromatic repeat arrays using two independent methods and identified significant polymorphism among them, with variation by as much as a factor of two in the centromeric 180 bp repeat, in the 45S rDNA arrays and in the Athila retroelements. In the accession with highest genome size as measured by flow cytometry, Loh-0, we found more than a two-fold increase in 5S RNA gene copies relative to Col-0; results from fluorescence *in situ* hybridization with 5S probes were consistent with the existence of size polymorphism between Loh-0 and Col-0 at the 5S loci. Comparative genomic hybridization results of Loh-0 and Col-0 did not support contiguous variation in copy number of protein-coding genes on the scale needed to explain their observed genome size difference. We developed a computational data model to test whether the variation we measured in the repeat fractions could account for the different genome sizes determined with flow cytometry, and found that this proposed relationship could account for about 50% of the variance in genome size among the accessions.

**Conclusion:**

Our analyses are consistent with substantial repeat number polymorphism for 5S and 45S ribosomal genes among accession of *A. thaliana*. Differences are also suggested for centromeric and pericentromeric repeats. Our analysis also points to the difficulties in measuring the repeated fraction of the genome and suggests that independent validation of genome size should be sought in addition to flow cytometric measurements.

## Background

The fundamental mechanisms that generate and shape genomic diversity – mutation, recombination, selection and drift – were well known before the genomic era. Despite advances, the variation of a eukaryote species' genome from individual to individual is still not well understood. A significant source of intraspecific diversity, variation in the copy number of genomic elements (Copy Number Variation, CNV) is defined [1] as deletions or duplications of any genomic elements, except transposons, greater than one thousand base pairs (bp). Emerging research suggests that genic CNV contributes to major changes in chromosomal organization and content between species, and disease in humans [1-4]. A number of methods have become available for detecting CNV, all facilitated by the availability of sequence information derived from analysis of the single or low copy fraction of the genome.

Heterochromatic repeats form a second genomic component subject to variation. No consistent term is in use to define copy number variation in transposons, transposon-related, centromeric and ribosomal repeats, which make up a considerable portion of eukaryotic genomes and are typically in heterochromatin [5]. To facilitate discussion, we will designate this latter type of variation as Repeat Number Variation (RNV). RNV can arise rapidly [6,7]. The significance of RNV is unclear – in the human population RNV has been reported both as general with no effect, and associated with disease [8-10]. Change in ribosomal RNA genes (rDNA) have been reported in plants [11-13].

Although several cases of repeat variations have been documented [14], RNV is harder to characterize than CNV. The larger repeat rich sequences of the genome cannot be tiled into contigs for physical mapping without ambiguity, due to their repetitive nature, and gaps of uncertain but megabase size persist in the sequenced genomes' repeats, including the human, in particular in centromeres [15,16]. For that reason major repeats have been excluded from the definition of a sequenced genome [17].

The uncertainty in the repeated component is illustrated by the status of the nuclear genome of the model organism Arabidopsis, one of the smallest in the vascular plants. The initial *Arabidopsis thaliana *genome sequence was announced by the Arabidopsis Genome Initiative (AGI) [18] in 2000, with the 1C (haploid, or single complement) genome estimated to be 125 million base pairs (Mbp); 115 Mbp had been sequenced, with work continuing on the centromeres and 5S rDNA. Subtelomeric rDNA arrays on chromosomes 2 and 4 [19] were not sequenced. The centromere structure and composition was explored by several groups. Work with pulsed field electrophoresis of the 180 bp centromeric repeat [20] was followed by its genetic mapping [21]; both better established its aggregate size and location on the chromosomes. A karyotype developed using FISH [22] with this repeat and a component of the pericentromeric Athila retrotransposon further refined the centromeric regions; the AGI sequence data and use of FISH [23] enabled more detailed elucidation of structure and chromatin status of the centromeres. The sizes of all 5 centromeres were assessed through partial sequencing and physical mapping [24-26] leading to an estimated size of 27 Mbp, three times the initial AGI estimate of 7 to 8 Mbp, and placing the total genome size near 146 Mbp. These conclusions were supported by the work of Bennett *et al*. [27]; Table 1 presents this changing understanding of the Arabidopsis genome size.

**Table 1:** Three estimates for the size of the *Arabidopsis thaliana *genome

Sequence class	AGI (2000)	Data source	Hosouchi *et al*. (2002)	Data source	Bennett *et al*. (2003)	Data source
Genes	51	S	--		--	
Intergenic DNA	59	S	--		--	
Centromeres	8	L	27	P	28	L
Distal rDNA	7	L	--		10 to 12	L
**Estimated total**	**125**		**146**		**147**	

Units are millions of base pairs (Mbp).

Data sources are S: Sequencing, L: Literature, P: Physical mapping.

Even with this imprecision in the repeated fraction the *Arabidopsis thaliana *nuclear genome is one of the best-characterized eukaryotic genomes, and provides an opportunity to better understand RNV in plants. A recent survey of Arabidopsis accessions through flow cytometry suggested variation in genome size [28]; it was not determined whether RNV or CNV was associated with these changes. Additionally, we do not know whether the differences detected by flow cytometry, which is based on the fluorescence of DNA-bound dye, reflect fluctuations in DNA content [29] or other differences in the status of the nuclear genome. For example, chromatin status significantly affects cytometric fluorescence measurements [30].

To explore RNV in the *Arabidopsis thaliana *genome, we measured the major repeats in several accessions by two different techniques. We documented considerable variation, particularly in the 5S ribosomal genes. Interestingly, the estimates of genome size inferred from repeat variation could only be fitted partially to measurements of total genomic size estimated by flow cytometry of nuclei. Comparative genomic hybridization of the Col-0 and Loh-0 accessions displayed CNV, but the observed variation could not account for the observed large differences in flow cytometric fluorescence of their nuclei.

## Results and discussion

### qPCR measurements of the major repeats

We used quantitative PCR (qPCR) to measure the amount of five major heterochromatic repeats in each of five accessions (Br-0, Is-0, Loh-0, Ta-0, TAMM-2), relative to the Col-0 plant's genome, which we used as a comparison standard in all assays. The sequences assayed are the 180 base pair centromeric repeat (CEN), fragments of the 18S and 25S ribosomal RNA genes, ORF1 of the high copy number pericentromeric Athila transposable element, and the 5S RNA gene. In the most recent (TIGR5) Arabidopsis genome there are 519 pericentromeric Athila genes totaling 1.6 Mbp. The 5S arrays [31] are only partly sequenced; their aggregate size is approximately 1 Mbp, updating the estimate of Campell *et al*. [32] to a 150 Mbp Arabidopsis genome.

Measurements of the relative amount of the major heterochromatic repeats in the five accessions are presented in Table 2. We assayed one individual in each accession by both quantitative PCR and nylon filter array hybridization, and assayed an additional individual, a sibling, in each accession using only qPCR.

**Table 2:** Measured size of heterochromatic repeat measurements in five *A. thaliana *accessions

Accession	Ind.	18S	SE	25S	SE	5S	SE	CEN	SE	Athila	SE
Ta-0	A	0.84	9	0.84	9	0.68	6	0.90	8	1.27	10
Ta-0	A	**0.60**	**12**	**0.87**	**12**	**1.07**	**5**	**1.29**	**4**	**2.61**	**11**
Ta-0	B	**0.79**	**11**	**0.70**	**19**	**0.62**	**16**	**1.27**	**11**	**2.61**	**13**
Br-0	A	0.85	6	0.85	6	1.15	10	0.84	7	1.05	13
Br-0	A	**1.09**	**10**	**1.18**	**15**	**1.75**	**4**	**1.10**	**9**	**1.40**	**11**
Br-0	B	**1.04**	**8**	**1.13**	**13**	**1.71**	**6**	**1.67**	**11**	**2.12**	**15**
Is-0	A	1.61	12	1.61	12	1.75	7	0.78	5	1.54	11
Is-0	A	**1.32**	**16**	**1.63**	**21**	**1.82**	**4**	**0.87**	**8**	**2.57**	**12**
Is-0	B	**1.38**	**10**	**1.50**	**13**	**1.40**	**8**	**0.95**	**12**	**2.17**	**14**
TAMM-2	A	0.59	6	0.59	6	0.89	8	0.76	8	0.92	9
TAMM-2	A	**0.71**	**11**	**1.02**	**8**	**1.15**	**4**	**1.60**	**8**	**1.40**	**11**
TAMM-2	B	**0.77**	**12**	**0.90**	**20**	**0.84**	**20**	**2.21**	**10**	**1.65**	**13**
Loh-0	A	1.68	6	1.68	6	2.28	9	0.54	5	1.10	15
Loh-0	A	**0.99**	**9**	**1.00**	**8**	**2.61**	**4**	**0.98**	**9**	**1.04**	**12**
Loh-0	B	**1.04**	**10**	**0.90**	**20**	**2.82**	**9**	**1.26**	**11**	**1.10**	**14**

Units are based on the Col standard (Col-0 = 1). Repeats in individual (A) were assayed using both filter array genomic hybridization and qPCR, its sibling (B) was measured with qPCR only. Filter values are presented in regular font, **qPCR in bold**. In the filter arrays, probes for the 18S and 25S subunits of the 45S rDNA gene were pooled; the same value is presented for each subunit. The number of observations for each value given is 24 for the filter assays and averages 12 for qPCR. Ind.: Individual.

SE: Propagated standard error of the mean, presented as percent of mean value. For measurement and error propagation details see Methods.

**Figure 1:**
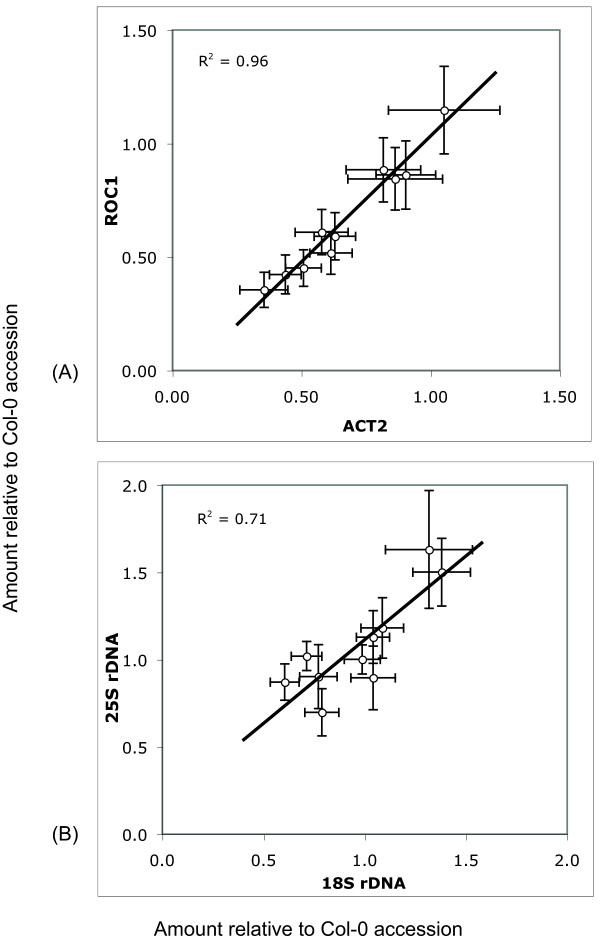
**Scatterplot comparisons of independent qPCR measurements**. Scatterplot comparisons of independent qPCR measurements; all values are relative to the amount in the Col-0 standard. (A) DNA concentration in ten samples as determined by separate qPCR of two singlecopy genes, ROC1 and ACT2. The linear regression between the two sets accounts for 96% of their variance (p-value ~10^6^). (B) Copy number of the of 45S RNA gene's 18S and 25S subunits, measured separately, from Table 2. Here linear regression accounts for 71% of their variance (p-value < 0.01). Horizontal and vertical bars present the standard error of the mean.

To achieve accuracy it was important to measure the input template DNA. Although we employed careful concentration measurements (see Methods), we decided to standardize our qPCR measurements using the single copy genes *ROC1 *and *ACT2*. Figure 1 panel (A) illustrates the relationship between the relative copy number of these two standards for the different input templates. The strong correlation (r^2 ^= 0.96) validated their use and indicate that they have balanced copy number in the accessions studied (we assume one per haploid genome); at the same time the results document the capability of qPCR to precisely measure template amounts. We also assayed the 18S and 25S subcomponents of the 45S repeat separately to assess the utility of the method in our study: their RNV among accessions should be identical. Panel (B) presents Table 2's qPCR results for the ribosomal RNA genes; linear regression between the separate subcomponents gives a coefficient of determination r^2 ^= 0.71 (p-value = 0.002), indicating good agreement.

The qPCR assays (Table 2) reveal the presence of broad polymorphism in copy number of the repeats; the measured amounts of the centromeric repeat, the pericentromeric transposable element Athila, and 45S rDNA vary by over a factor of two, and the 5S rDNA cluster by a factor of four, between the lowest and highest.

### Nylon filter array hybridization measurements of the major repeats

**Figure 2:**
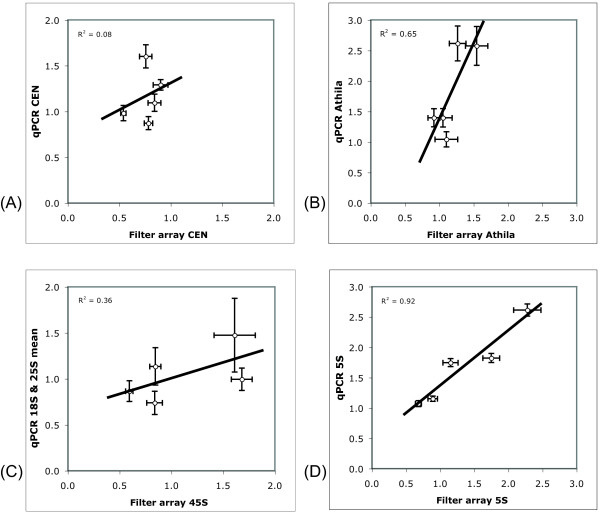
**Comparison of filter array and qPCR repeat copy number measurements**. Comparison of filter array and qPCR repeat copy number measurements (A) 180 bp centromeric repeat (B) Transposable element Athila (C) 45S rDNA (D) 5S rDNA. Horizontal and vertical bars present the standard error of the mean. Line segments present the least squares fit between the two sets of values; the coefficient of determination (R^2^) is given for each panel.

Filter arrays can provide an alternative measurement of the copy number of a repeat. We deposited each target sequence in multiple slots of the filter array to provide repeated measurements per array (see Methods for details). Labeled probes were hybridized to the filter array of genomic DNA and detected via fluorescence. We pooled the 18S and 25S RNA genes' probes in our filter measurements; these and the measurements of the other major repeats are presented in Table 2. The degree of variation in each repeat is consistent with that observed by the qPCR analysis. Figure 2 illustrates the relationship between the measured repeat amounts for the two methods. The relationship is excellent for 5S, good for Athila, mediocre for 45S, and bad for CEN. The discrepancies may be explained by the different specificity of the qPCR and filter array: the first depends on near perfect identity between primers and the corresponding target sequences, the second is more tolerant of variation between labeled DNA and the target on the filter array. This difference is consistent with the poor relationship displayed by the measures of the CEN repeats, which are known to vary [33]. It does not explain, however, the discrepancy in the measurements of the 45S rDNA, which is highly conserved within genomes. In conclusion, this comparison suggests confidence in the 5S measurements, but also illustrates the difficulty of measuring the repeats. Both methods may perform suboptimally in the pericentromeric Athila sections, which have experienced multiple transposition events into those sequences.

### FISH analysis

**Figure 3:**
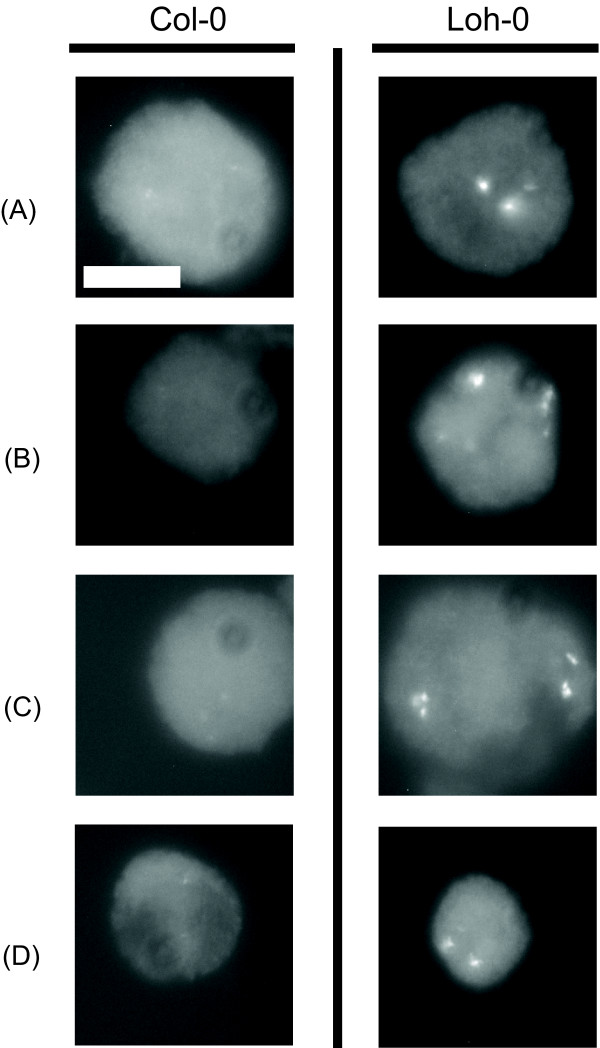
**Fluorescence intensity comparison between the 5S rDNA arrays in the sequenced accessions Col-0 and Loh-0**. Fluorescence intensity comparison between the 5S rDNA arrays in the sequenced accession Col-0 and Loh-0, the accession with the largest measured genome size. The images are unmanipulated FISH photomicrographs of meiotic pollen cells from anther squashes. The size marker is 10 μm in all images. (A) Col-0 and Loh-0 cells in pachytene of meiosis division 1 (M1). (B) M1 diplotene cells. (C) M1 anaphase cells. (D) Pollen microspores. The analysis is detailed in Methods.

The 3 to 4 fold measured variation in 5S rDNA repeat is substantial. To validate these observations, we prepared cytological slide mounts of anthers from the reference accession Col-0 and the accession with the highest measured 5S rDNA copy number, Loh-0. To achieve uniformity of hybridization we mounted and hybridized samples from both ecotypes side by side on the same slide. We probed the nuclei with a fluorescently labeled fragment of the 5S gene. We omitted protease treatment of the nuclei, a step that usually enhances hybridization efficiency, to achieve the best dynamic response. Pictures were taken at similar settings and representative raw images (not adjusted digitally in any way such as for contrast or exposure) from the assays are given in Figure 3. The panels present images of meiotic pollen mother cells in the two accessions: note that the background fluorescence displayed by the nucleoplasm is comparable in the two samples. The set of Loh-0 5S rDNA signals was scored as significantly brighter than Col-0 (chi-squared p-value < 0.005) by four observers; 20 Col-0 and 22 Loh-0 cells were scored in each set. The *in situ *results demonstrated that the two accessions have a qualitatively different hybridization signal to the 5S rDNA probe, corroborating differences in the amount of the 5S repeat in the two accessions.

### Sibling variation

We measured the copy number of each 18S and 25S rRNA gene in siblings of each accession (Table 2). For both subunits the difference in measured copy number between siblings is less than the standard error of the mean. The qPCR assays identified larger differences in the other repeats between siblings than the average 10% in the 18S & 25S ribosomal RNA genes, with a mean difference of 24% in 5S rDNA, 21% in the 180 bp repeat, and 16% in Athila. While Arabidopsis is almost entirely a selfing plant and is expected to be homozygous, development of polymorphism in heterochromatin of inbred plants has been reported [34,35]. Overall the measured differences between siblings are a small fraction of that determined among the accessions; over repeated generations, however, drift in the copy number of these elements could contribute to large differences.

### Fluorescence measurements of nuclei by flow cytometry

We measured the fluorescence of propidium iodide stained nuclei of the sequenced accession Col-0. Using commercially-available alcohol-fixed chicken erythrocyte nuclei from Becton-Dickinson as the internal size standard, and taking the *Gallus gallus *1C genome size to be 1150 Mbp [36], we derived a size of 157 Mbp (0.160 picogram) for Col-0. This is close to the 163.7 Mbp measurement by Bennett *et al*. [27], which was based on the *Gallus *and additional standards, but 25% larger than the 125 Mbp estimated by the AGI [18]. Our estimate is also much lower than the 202 Mbp value estimated by Schmuths *et al*. [28] using *Raphanus sativus *(the cultivated radish) as an internal size standard (680 Mbp) [37].

**Figure 4:**
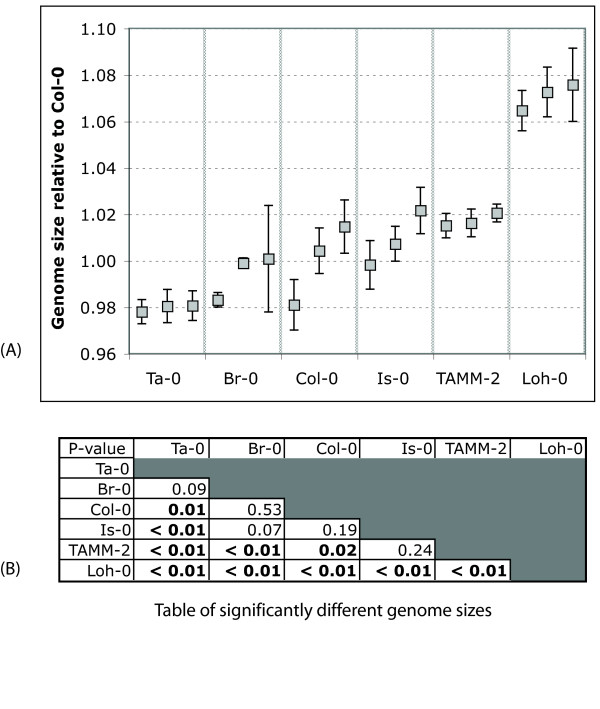
**Distribution of genome size measurements of five accessions in *Arabidopsis thaliana***. Distribution of genome size measurements of five accessions in *Arabidopsis thaliana*. Values given are relative to the average measured genome size of the sequenced accession Col-0. (A) Genome size of three individuals in all accessions, each assayed by flow cytometry four times over a period of a week. Error bars give the standard error of the mean of the four observations. (B) Table of ANOVA p-values, values in bold indicate accessions with significantly different genome size distributions, using the 12 measurements for each accession.

We tested the five accessions used in the repeat variation measurements for their nuclear fluorescence response by flow cytometry. The inferred genome sizes are presented in Figure 4(A) relative to Col-0. Two accessions, Ta-0 and Br-0, have mean measured genome size smaller than the sequenced accession Col-0, and three, Is-0, TAMM-2 and Loh-0, are larger. The fluorescent response of Loh-0 is consistent with a 15 Mb larger genome than Col-0.

Fig. 4(B) shows that measured differences in genome size between nearest neighbor accessions (in genome size) are not always significant. This could in part be due to the precision of the method and also in part to variation in genome size among siblings. Panel (A) shows that genome size variation in siblings is not significant for three accessions (Ta-0, TAMM-2, Loh-0), but is for the three others (Br-0, Col-0, Is-0). To determine whether that variation and the small mean differences between nearest neighbors are accurate will require further study. We selected these accessions for this study as they spanned the genome size range of the 22 in our initial survey of Arabidopsis. The study measurements were made using a separate set of individuals; survey results are available here in three additional files [See Additional files 1, 2 and 3].

### Comparative Genomic Hybridization assays

The unusually high nuclear fluorescence response displayed by Loh-0 suggested the possibility of large scale CNV in this accession. We wanted to determine, therefore, if Loh-0 had one or more segmental duplications of chromosomes. We employed comparative genomic hybridization (CGH) with spotted oligonucleotide gene microarrays to assay the copy number of genic sequences in Loh-0, compared with the sequenced Columbia accession (detailed in Methods). The microarray oligos are designed from known genes, EST sequences and predicted transcripts. A number of transposable elements (190 known transposon-related features), a class chiefly closely associated with centromeres and nearby sequences, are present on the array. While represented, this class of genes is not present in the quantity in our array data, especially on chromosomes 4 and 5, relative to their known presence in pericentromeric regions of the genome. This array in addition cannot assay the copy number of intergenic sequences or the centromere cores as both are absent from the set; neither are the ribosomal RNA genes represented.

**Figure 5:**
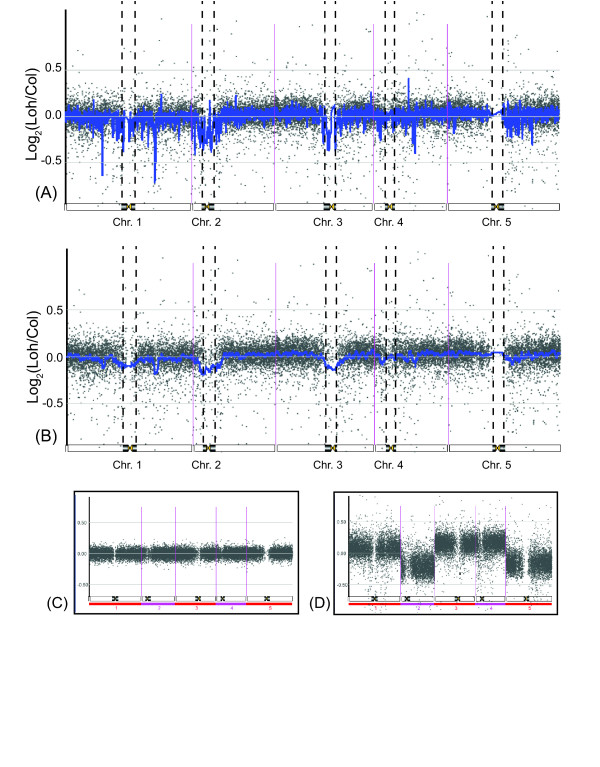
**Comparative Genomic Hybridization (CGH) microarray results**. Comparative Genomic Hybridization (CGH) microarray results. Individual values presented are the base 2 logarithm of the fluorescence ratios of two *Arabidopsis thaliana *genomic DNA samples hybridized to slide features. Features are ordered left to right by position on chromosomes 1 through 5. The constriction on each chromosome marks the location of the centromere, each arbitrarily one million base pairs (Mbp) wide in this diagram. Dark bars flanking centromeres mark pericentromeric regions where Athila retrotransposon loci are present in the sequenced genome. Panels (A) and (B) present the same values overlaid with a blue line presenting a 15-point running mean in panel (A) and a 101-point running mean in panel (B); values are the feature hybridization signals of accession Loh-0, relative to the sequenced accession Col-0. Note that the array was constructed using information from the sequenced Col-0 accession. Panel (C) presents the ratios of a CGH selfself hybridization assay with accession Col-0. Panel (D) values are of an aneuploid individual with three copies of chromosomes 1, 3 and 4 and two copies of chromosomes 2 and 5. Values are relative to feature signals from a diploid individual; from Henry *et al*. (2006), see this for additional information.

After quality control of the hybridization data, some 18,000 hybridized features remained for this analysis. Figure 5 presents the hybridization results. Values charted are the base 2 logarithm of Loh-0 feature intensities, relative to Columbia; see the caption for a detailed explanation. The suitability of this array system for CNV analysis are demonstrated in panels (C) and (D). The ratio observed with self versus self demonstrates the linear response of the hybridization ratio. In contrast, when a known aneuploid of Arabidopsis [38] is compared to the diploid, chromosomes present in three copies can be readily identified by the ratio of aneuploid/diploid hybridization. Therefore, segmental duplications or deletions that encompass more than several contiguous array features are readily detected. Such is case in the comparison of Loh-0 vs Col-0. Two regions whose microarray features display ratios consistent with deletion in Loh-0 are detected in the euchromatic arms that flank centromere 1. One, beginning between At1g24735 and At1g24938 and ending between At1g25220 and At1g25230, is centered at 8.8 Mbp for approximately 100,000 bp (or 0.1 Mbp). The other, beginning between At1g58480 and At1g59077 and ending between At1g59406 and At1g59520, is centered at 21.4 Mbp for approximately 200,000 bp (or 0.2 Mbp). A region on chromosome 4 encoding a cluster of putative resistance genes is present in higher copy number in Loh-0, consistent with expansion of these genes, beginning near At4g16845 and ending near At4g16980, centered at 8.47 Mbp for approximately 80,000 bp. Unequal crossing over between tandemly repeated resistance genes is known [39] to result in copy number variation.

In addition, a moving average of the ratio of several features dips in value close to the centromeres of chromosomes 1, 2, and 3. The pericentromeric region's ratios, as defined by the presence of Athila elements in the TIGR sequence, have a mean of 0.97. The same value for the chromosome arms is 1.02. This indicates that pericentromeric features in these chromosomes did not hybridize to Loh-0 DNA probably because the corresponding genes are either absent or diverged in this strain. Such degree of polymorphism is expected because the pericentromeric features are enriched in transposons and pseudogenes, whose loss or degeneration should be neutral and not selected against. The CGH centromeric trend cannot be taken to indicate that there is a net loss of pericentromeric genes in Loh-0 compared to Col-0. The array was constructed based on Col-0 sequence and it therefore cannot provide information on sequences that may be present in Loh-0 and absent in Col-0. We conclude that the analysis does not support the existence of large segmental duplication involving the known genes of Col-0.

### Modeling genome size variation

There is a discrepancy between the *A. thaliana *Col-0 genome size predicted by AGI's accounting of sequenced DNA (about 125 Mbp), and that inferred from flow cytometry (almost 160 Mbp). One possible explanation is that flow cytometry has a systemic bias. For example, a difference in condensation of chromatin between the internal *Gallus *standard and the test genome might perturb the measurement and produce a large error (~20%). The concentration of propidium iodide we used is supposed to minimize these effects [40]. Nonetheless, we tested the effect of chromatin remodeling by comparing individuals of the Landsberg *erecta *accession and its *ddm1 *mutant, finding only about 4 Mb mean difference, within the range exhibited by the wild-type individuals (data not shown). The *ddm1 *mutation introduces profound changes in chromatin state [41]; chromatin changes of the type observed in *ddm1 *mutants, could contribute to apparent genome size differences but are unlikely to be the main determinant of the Loh-0 to Col-0 difference.

An alternative hypothesis is that the repetitive fraction of the genome is different than estimated by the AGI. We developed a data model to assess whether our measured repeat fractions could account for the different genome sizes we determined with flow cytometry. The model first calculates the size of the variable genome in each individual as the size in Mbp of each of the heterochromatic elements in the sequenced Col-0 genome times the individual's qPCR-measured repeat amount relative to Col-0; the combined size of the basal genic and intergenic regions (108 Mbp) is added to give the total genome size. Given that the sequenced genome's heterochromatin repeat sizes are not known with precision, the model tests a series of sizes for each repeat, drawing on published size estimates to establish a range.

Because of the unsettled understanding of the size of the Arabidopsis genome, we determined separate sets of these values for Arabidopsis genome sizes of 130, 145, and 160 Mbp. As an example, assuming the true Col-0 genome size is 130 Mbp, the model alternately tries several sizes for each repeat in Col-0 in turn, then calculating the modeled genome size for each accession. For this the size of the repeat in each accession, relative to its size in Col-0 (from Table 2) is used. We designed a merit function [42] to assess agreement between the flow cytometry-measured and model-predicted genome sizes, and used it to identify Col-0 repeat sizes giving the best overall fit. Conceptually, the set of repeat sizes giving the smallest difference between the modeled and measured genome sizes is chosen. In the example, a 5S array size of 6 Mbp, along with the other repeat sizes for a 130 Mbp Col-0 genome, minimizes the error between modeled and measured genome sizes. We used only the qPCR results in this analysis.

**Figure 6:**
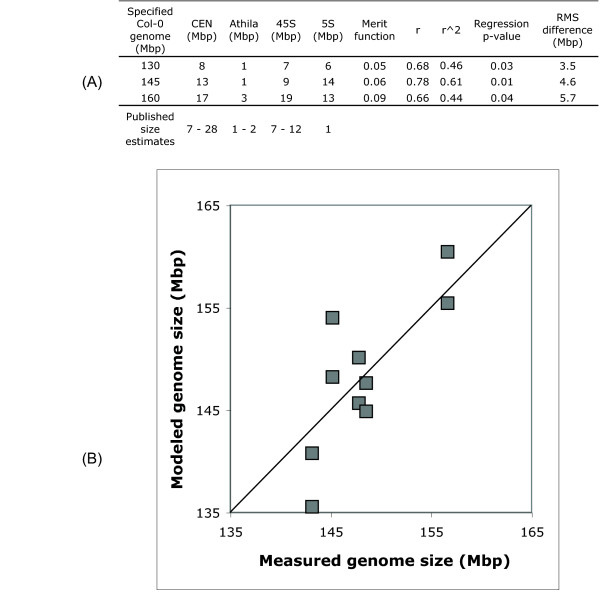
**The assayed accessions' genome sizes modeled as the sum of a basal genome plus repeats**. The assayed accessions' genome sizes modeled as the sum of a 108 Mbp sequenced basal genome plus four major repeats which vary in size among the accessions. As the absolute size of these repeats is not known we fit them to three possible totals for the *A. thaliana *genome. The model is detailed in the Methods section. (A) The modeled size of the repeats in the sequenced genome of Col-0 is given for each of these, along with measures of agreement between the modeled genome sizes and those determined with flow cytometry. Published estimates for the size of each repeat in Col-0 are listed in the bottom row. (B) Best overall fit of modeled genome size versus the measured genome size for each individual, a 145 Mbp Col-0 genome, using all measurements. The line of perfect agreement between the modeled and measured genomes is drawn.

We found (Figure 6) that variation in the four large repeat arrays we assayed account for up to 61 percent of the variance in measured genome size among the accessions. A Col-0 genome of 145 Mbp generates the best overall fit to the repeat data, and modeled repeat sizes fall within published estimates, except for the 5S array. The accession with the largest measured genome, Loh-0, could challenge the model due to its extreme measured genome size and pattern of variation. When omitting this accession from the model the assayed differences in four repeats explain up to 49 percent of measured genome size variation, and the 5S array is put at 2 Mbp in a 145 Mbp genome. The modeling work indicates that about half of the genome size variation suggested by flow cytometry can be validated by measuring the four major repeats of the *Arabidopsis thaliana *genome. Discrepancies between the measured and modeled genome sizes may result from variation in repeats present but not modeled, and uncertainty in the measured repeat fraction sizes.

## Conclusion

Our analyses are consistent with substantial repeat number polymorphism for 5S and 45S ribosomal genes among accession of *A. thaliana*. Differences are also suggested for centromeric and pericentromeric repeats. The largest difference for 5S ribosomal genes from the Col-0 standard was observed in accession Loh-0, which is also the most extreme of those tested in propidium iodide fluorescence of nuclei. As over 200 repeat families have been identified in Arabidopsis [43], our study is not exhaustive. Expansion and contraction in these, and creation of new families in individual accessions, will likely continue to contribute to divergence within the species and might underlie what we observed in Loh-0.

Our analysis also points to the difficulties in measuring the repeated fraction of the genome and suggests that independent validation of genome size should be sought in addition to flow cytometric measurements. Proper accounting of the repeated genomic fraction may require nonbiased parallel shotgun sequencing methods; see [44] as an example of recent advances.

## Methods

### Arabidopsis accessions and growth conditions

We acquired Arabidopsis accession seed from the Arabidopsis Biological Resource Center (ABRC) at Ohio State University, and from Prof. Magnus Nordborg at the University of Southern California. The accessions reported here are: ABRC stock numbers CS1548 (Ta-0), CS1240 (Is-0), CS1350 (Loh-0), and from Prof. Nordborg 9A Br-0 A (Br-0), 8F Col-0 A (Col-0), 6A TAMM-2 A (TAMM-2). Seed were sown directly on wet potting soil in 2 inch pots, maintained in the dark at 4°C for four days, and moved to a 22°C growth room where the plants germinated and were grown under fluorescent lights with 16 hours light and 8 hours dark per day. The *ddm1 *homozygote mutants used in comparison with the Landsberg *erecta *accession were in their second or third generation of homozygosity.

### Genome size determination with flow cytometry

Genome size measurements were made at the Cell Analysis Facility of the Department of Immunology, University of Washington; a Becton-Dickinson FACScan flow cytometer with 488 nm argon laser was used. Linearity of instrument response to DNA content were assayed using aggregated chicken erythrocyte nuclei.

Sample preparation was as follows. Stained nuclei: 100–300 mg of leaves were collected and stored temporarily in a petri dish on ice. Chopping buffer (1.5 ml) was added to the dish, and leaves chopped with a razor blade, mixing until a paste was formed, 2 to 4 minutes. Liquid was collected and aspirated with a syringe; filter holder (Millipore Swinnex 25 mm) attached with 30 μm filter fitted inside (Small Parts Inc CMN30 monofilament cloth), and pressed through the filter into a microfuge tube. Tubes were spun at 500 × g for 7 minutes; supernatant discarded and 3 μl of the internal standard added, chicken erythrocyte nuclei (Becton-Dickinson DNA QC particles, Cat. No. 349523, or BioSure chicken erythrocyte nuclei singlets, Cat. No. 1013), and nuclei resuspended in 700 μl staining solution. Samples were capped and stored above ice at least 2 hours prior to evaluating DNA content, and protected from light. Chopping buffer: modified from Bino *et al*. [45], 15 mM HEPES, 1 mM EDTA, 80 mM KCl, 20 mM NaCl, 300 mM sucrose, 0.20% TritonX, 0.5 mM spermine, 0.10% β-mercaptoethanol (BME). Buffer without BME may be stored at 4°C indefinitely; BME is added just before use. Staining buffer: 50 μg/ml of the fluorochrome propidium iodide (PI) and 50 μg/ml RNAse A was added to chopping buffer. PI is a potential mutagen and handled accordingly.

We note that the absolute value of the chicken (*Gallus gallus*) genome size is uncertain. Resolution of the uncertainty in the repeated fraction – responsible for the uncertainty in genome size in both Gallus and Arabidopsis – requires an independent method, other than flow cytometry. The Gallus standard can be expected to be exact for the relative comparison of Arabidopsis accessions.

### DNA extraction

Plant DNA was extracted from 1 gm rosette leaves, ground for several minutes in a mortar, initially with a small amount of liquid nitrogen to facilitate reducing the leaves to powder. Plant extraction buffer (150 mM Tris pH 8.0, 50 mM EDTA, 500 mM NaCl, 0.7% SDS, 50 μg/ml Proteinase K, 50 μg/ml DNAse-free RNAse A) was added to a total volume of 8 ml during grinding. The sample was filtered through Miracloth and heated in round-bottom tubes in a water bath at 55°C for 3–5 hours; 4 ml saturated NaCl was mixed in each tube and spun in a preparatory centrifuge at 7,000 × g for 20 minutes. The supernatant was divided into 2 tubes and 7 ml 85% isopropanol added and mixed by inverting; supernatant was discarded after spinning again for 10 minutes, the pellet washed twice in 70% ethanol, and air-dried for 10 minutes. The pellet was resuspended in 1 ml TE and transferred to a 1.5 ml tube; 1 μl 25 mg/ml RNAse A added and incubated at 37°C for one hour. The procedure was completed with phenol extraction and ethanol precipitation and washing, and after air-drying the sample was resuspended in TE and frozen at -20°C.

### Filter array hybridization

Biodyne nylon transfer membranes were cut to fit in the Bio-Rad Bio-Dot SF blotting apparatus with 48 wells; each well is 7 × 0.75 mm. Membranes were loaded with genomic DNA extracted from 2 individuals, one the single standard loaded on each membrane, the second a test plant; before loading on blots the DNA was fragmented by passage through a  narrow gage needle.  DNA concentration was quantified using a Turner fluorometer with SYBR green dye from Molecular Probes and a lambda-phage DNA standard; when it became available sample DNA concentration was reassayed with a Perkin Elmer Victor3 V plate reader. Before loading, DNA extracts were heated to 100°C in boiling water for 10 minutes, immediately cooled on ice, and diluted to 1 ng/μl in 0.4 M NaOH. Each sample was loaded in 8 slots in one of 3 amounts, 100, 125 or 150 ng for a total of 24 slots per plant distributed across the array to assay linearity of fluorescence with hybridization. The loaded DNA was neutralized by floating the membrane on 100 mM Tris pH 8, cross-linked to the membrane with a UV Stratalinker and allowed to air dry before use.

The Amersham Biosciences AlkPhos Direct Labeling Enhanced Chemifluorescence System was used to fluorescently label the DNA probes, hybridize probes to membrane-bound genomic DNA, and develop the hybridized labeled probe according to the manufacturer's instructions. Fluorescence was excited and detected with the UVP Epichemi3 Darkroom/Benchtop UV Transilluminator with filter set to 515–570 nm, and membrane images captured with a digital camera. Signal intensity was quantified with the ImageJ open source gel blot analysis software available from the Research Services Branch of the U.S. National Institutes of Health. Blots were stripped of hybridized probe according to the manufacturer's instructions, stored in 100 mM Tris pH 8 at 4°C and reused.

DNA probes from 120 to 700 bp in length were generated using the PCR of DNA extracted from Arabidopsis accession Columbia-0 with the following primers: the 180 bp centromeric repeat (5'-CAT GGT GTA GCC AAA GTC CAT A-3' and 5'-GCT TTG AGA AGC AAG AAG AAG G-3'; ORF1 of the Athila retrotransposon was amplified using degenerate primers and a touchdown thermocycler program as described in Josefsson *et al*. [46]. The 5S rDNA gene primers were (5'-GAT GCG ATC ATA CCA GCA CT-3' and 5'-GGA TGC AAC ACG AGG ACT TC-3'), 18S rDNA gene (5'-GCA TTT GCC AAG GAT GTT TT-3' and 5'-GTA CAA AGG GCA GGG ACG TA-3'), and 25S rDNA gene (5'-AGA ACC CAC AAA GGG TGT TG-3' and 5'-TCC CTT GCC TAC ATT GTT CC-3').

The amount of heterochromatic repeat in each accession relative to the single standard was calculated as the ratio of the accession's mean value (A) on a blot divided by the standard's (B). To estimate uncertainty in the results, the standard error of each measured value (ΔA, ΔB) was used; the relationship of the final parameter to the measured variables was used to propagate standard errors. For a function of two variables the uncertainty is ΔF(x, y) = ((F_x _Δx)^2 ^+ (F_y _Δy)^2^)^1/2^, where the subscripts indicate partial derivatives. In the filter arrays with F(A, B) = A/B, the fractional standard error is ΔF/F = ((ΔA/A)^2 ^+ (ΔB/B)^2^)^1/2^.

### Quantitative PCR

Quantitative PCR reactions were run in 96-well plates in a Chromo4 Continuous Fluorescence Detector and Thermocycler from MJ Research, Inc; initial data analysis was made using the Opticon Monitor software from the same company. The individual DNA samples used in the filter assays were also used in these assays. Replicates (from 6 to 12 of each sample and amplicon) were loaded distributed across a plate, using DNA in 3 amounts, 1.00, 1.25 and 1.50 times a basal loading, in order to assess linearity of amplification and detection. DNA extracts used in qPCR reactions were diluted 25× in water before use; the fluorophore used was SYBR green.

Reaction volumes were 20 μl with the following reagents (per reaction: 11 μl water, 1.6 μl 2.5-mM dNTPs, 0.2 μl 20-μM primers, 0.05 μl 100×-SYBR green, 0.2 μl 5 U/μl-*Taq *polymerase, 2 μl 10×-buffer, and 5 μl genomic DNA, approximately 5 ng/μl). The thermocycler protocol was (94° for 120 seconds, then cycle 40 times: 94° for 20 seconds, 57° for 20 seconds, 72° for 30 seconds; using a heated lid at 100°). A melting curve was generated for each reaction product to test for multiple amplification products. Amplicon template quantities were measured using a threshold cycle (C_t_) method [47,48]. See Larionov *et al*. [49] for a discussion of error analysis and reduction. Briefly, for each amplicon, a dsDNA fluorescence value was selected where all accessions' templates had been amplified to the same copy number; the threshold cycle where this occurred was recorded for each accession. Relative amounts of initial template quantity Q_o _were calculated with the relationship Q_o _= A^-Ct ^where A is the cycle amplification factor. Replicates were averaged to provide a single value for analysis. Estimates of uncertainty used the standard error of the individual estimates of A and C_t _with the errors propagated as in the filter arrays. The propagated fractional standard error is ΔQ_o_/Q_o _= ((C_t_/A)^2 ^(ΔA)^2 ^+ (ln(A))^2 ^(ΔC_t_)^2^)^1/2^.

The copy number of the assayed repeats in each sample's genome was measured as the ratio of template amount of the repeat to the template amount of single copy gene amplicons. Amplification products were from 120 to 300 bp long. The following primers were used: for the 180 bp centromeric repeat (5'-CCG TAT GAG TCT TTG GCT TTG-3' and 5'-TTG GTT AGT GTT TTG GAG TCG-3'); probes of the retroelement Athila were derived from *A. thaliana *sequences amplified with degenerate primers and cloned [46]. Representative clones were aligned to identify conserved sequences from which these primers were designed; Athila ORF1 (5'-TTT CTC ACT AGG GGA TAA AGC TCA-3' and 5'-CAA TCT AGC CGT TCT TGA GTT AGA-3'). Primers for the 5S rDNA gene were as in the membrane hybridization; for the18S rDNA gene (5'-CCT GCG GCT TAA TTT GAC TC-3' and 5'-GAC AAA TCG CTC CAC CAA CT-3'), and 25S rDNA gene (5'-CGC GAG TTC TAT CGG GTA AA-3' and 5'-CAC TTG GAG CTC TCG ATT CC-3'). Single copy genes used were actin (ACT2, At3g18780) (5'-TGC CAA TCT ACG AGG GTT TC-3' and 5'-TTA CAA TTT CCC GCT CTG CT-3), and cyclophilin (ROC1, At4g38740) (5'-TCA AGC CAA TCG GTC TTC AC-3' and 5'-CGA TCT ACG GGA GCA AGT TC-3').

We assayed DNA extract genome copy number using qPCR of two single copy genes and independently confirmed those results with excellent agreement (r = 0.97, data not shown) using a plate reader and the fluorescent dye SYBR green to measure DNA concentration.

### Fluorescent In Situ Hybridization (FISH)

The plant material was prepared as in Comai *et al*. [50], with the following changes: the *A. thaliana *180 bp centromeric repeat probe fluorescent dye was fluorescein-12-dUTP (FITC, Roche 1373242) and the 5S rDNA array probe fluorescent dye was tetramethyl-rhodamine-5-dUTP (Roche 1534378). Probes were amplified with the primers identified in the filter array method. Prepared slides were visualized using the Nikon Microphot-FX fluorescent microscope; images were captured with the Qimaging Retiga 1300 monochrome 10 bit digital CCD camera and processed with the Improvision Openlab image analysis software, V4.0.4. Camera exposure times were chosen to maximize image clarity without saturation of pixels. Photographs were taken, and reviewed and cropped using Adobe Photoshop; no additional image enhancement was performed.

To assess relative amounts of the 5S rDNA repeat in the Col-0 and Loh-0 pollen mother cells, anther squashes of both accessions were prepared side-by-side on slides and probed. In the scoring process, 20 Col-0 and 22 Loh-0 images were randomly presented in gray-scale using the JPEGDeux open source slideshow application. The scoring individual identified the 5S intensity in each image as either plus or minus without knowing the accession (blind scoring). Four people independently scored the set of 42 images, and all identified the Loh-0 accessions as significantly brighter (chi-squared p-value < 0.005). Combined scores for each accession are 5% plus for Col-0 (plus and minus = 4 and 76), and 66% plus for Loh-0 (plus and minus = 59 and 29).

5S loci are present on chromosomes 3, 4 and 5 in Col-0 but the number and localization of 5S loci in Loh-0 are not known. We reviewed the Loh-0 FISH images to count 5S loci and identified up to three spots. We believe that the number of loci in the accession is constant – that is, three, the same as in the Col-0 accession – and in several images with fewer than three spots, in those slides the loci lie one over another.

### Comparative genomic hybridization

The Operon 26,000 oligo set was used to print microarrays in the Fred Hutchinson Cancer Research Center facility. Feature density in the chromosome arms is 1 per 6,000 bp and in the pericentromeric regions 1 per 14,000 bp; on chromosome 5 the near-centromere value is 1 per 30,000 bp. Ratios measuring the relative amount of 26,090 70-mer sequences in two accessions' genomes were derived in the following way. For each of the two samples to be combined and assayed, 300 ng of unfragmented genomic DNA were labeled with either Cy3-dUTP or Cy5-dUTP (Amersham Cat. PA53022 or PA55022) using the Invitrogen Bioprime Array CGH Genomic Labeling System Cat. 18095-12, according to the manufacturer's instructions.

The Cy3 and Cy5-labeled samples of each accession were then combined and purified using the Qiagen QIAquick PCR Purification Kit Cat. 28104, according to the manufacturer's instructions. Yeast tRNA (Invitrogen Cat. 15401-029) was added to the labeled DNA at a final concentration of 0.5 mg/ml and SSC at 3× final concentration in 120 μl total volume. Each sample pair was also dye-swap labeled.

The labeled DNA samples were hybridized to a spotted microarray of the Operon Arabidopsis Genome Oligo Set Version 1.0 and washed and scanned at the DNA Array Facility of the Fred Hutchinson Cancer Research Center. Image conversion was done with GenePix Pro 6.0 and these data analyzed with the TIGR open source Microarray Data Analysis System, MIDAS [51]. Dye-swap pairs were filtered to discard features with signal-to-noise ratio less than two, and LOWESS normalized separately before being combined. Dye-swap consistency was checked, integrated feature intensities of each channel were written, and ratios of relative intensity calculated. Note that the normalization applied to the ratios in order to correct for microarray block and dye intensity-dependent effects constrains the global mean to exactly one.

Further analysis of the data was carried out using the open source application CGH-Explorer, available from the Department of Informatics, University of Oslo [52], and the Microsoft Excel spreadsheet application.

### Modeling heterochromatin contributions to genome size

We developed a numerical data model to provide an estimate of the absolute contribution of each of the heterochromatic repeats to the sequenced Arabidopsis genome. The model minimizes the difference between the set of flow cytometry-determined genome sizes and the set of genome sizes calculated from the repeat sizes measured by qPCR plus a basal, constant genome component. We estimated the last element at 108 Mbp, taking the sequenced amount of 115 Mbp [18], subtracting a sequenced 5 Mbp reported there from the centromeres, and 1 Mbp apiece for the Athila TE and 5S rDNA repeats. The 45S rDNA arrays were not sequenced. The modeled relationship between the two sets is **Y **= m**X **+ b where the symbol meanings are:

**Y **vector of measured genome sizes, with element y_i _for the i^th ^individual (Mbp)

**X **vector of the summed repeat sizes with element x_i _for the i^th ^individual (Mbp)

c_j _size of the j^th ^repeat in the sequenced Col-0 accession (Mbp)

w_ij _fractional amount of the j^th ^repeat in the i^th ^individual, relative to Col-0

x_i _repeat total in an individual: x_i _= c_j_w_ij _with j summed over all repeats (Mbp)

m a scaling factor between repeat size and genome size measurements

b the basal, unvarying genome component (108 Mbp)

The relationship is a simple linear one: we expect the amount of polymorphic repeats and the basal component to add up to the measured genome size of an individual. The scaling factor (m) is present to correct for any linear distortion in the response of the qPCR system to difference in repeat amount – if for example a 30% difference is measured as 40%. Ideally m = 1, but is not be assumed to be.

The computational model is written in Perl; it first reads for each assayed individual its ID, the measured genome size of the accession and the sizes of the centromeric, Athila, and 45S and 5S rDNA arrays relative to the comparison standard Col-0 individual. We use the accession mean rather than the individual measured genome size as separate flow cytometry measurements of any individual appear to be randomly distributed around the accession mean; we average the 18S and 25S qPCR repeat size values to form a single 45S measurement.

Numerical values for repeats in Mbp are calculated for each individual by summing the products of the size of each repeat in that individual (relative to the Col-0 standard) times the size of the repeat in the sequenced accession Col-0. The latter values are not precisely known as the repeats are unsequenced. The model sequentially assigns values to each repeat in Col-0 from a range of potential sizes; the ranges are, for the centromeric repeat 8–30 Mbp, Athila transposon 1–10 Mbp, 45S rDNA 7–20 Mbp and the 5S rDNA 1–15 Mbp. Most combinations of four repeat and basal genome size do not sum to the assigned Col-0 genome size and are discarded; the Col-0 total genome size is specified as a particular value for each model run. A merit function for each of the combinations passing this screen is calculated in the following way: the genome size of each individual is summed from its component parts; the standard deviation of the difference between the measured and calculated genome sizes (the RMS error) is then divided by the correlation coefficient between the two sets of values. If the correlation is less than 0.1 the combination is discarded. Optimal values of the scaling factor (m) are also assayed. The Perl script writes out the merit function value, associated repeat and basal genome sizes and scaling factor to a file and proceeds with the next combination. Combinations with the smallest merit function are reviewed.

### Statistical analyses

Statistical tests were evaluated using Microsoft Excel and its data analysis tools. The p-value reported for linear regressions is the regression tool ANOVA table's F-test results (Significance F). The 5S rDNA chi-squared test results were assessed using a table of critical values for the chi-squared distribution; the ANOVA p-value is that calculated by Excel's single factor ANOVA tool.

## Authors' contributions

JD designed and carried out the qPCR, filter array, flow cytometry and CGH assays and analyses, and drafted the manuscript. AT carried out the FISH assays and participated in their interpretation and analysis. LC conceived of the study, and participated in its design and coordination and helped to draft the manuscript. All authors read and approved the final manuscript.

## Supplementary Material

Additional file 1 Genome size measurements. A discussion of our genome size survey in 22 *A. thaliana *accessions. Click here for file

Additional file 2 Survey of genome size variation in *A. thaliana *determined by flow cytometry. A table of the genome size variation we measured in Arabidopsis. Click here for file

Additional file 3 Flow cytometry-determined average genome size and standard deviation (Mbp) of 22 Arabidopsis accessions. A figure presenting the mean genome size we measured in 22 Arabidopsis accessions. Click here for file
