# Supplementary material for: Large-scale polymorphism of heterochromatic repeats in the DNA of Arabidopsis thaliana
Source: BMC Plant Biol. 2007 Aug 16;7:44. doi: 10.1186/1471-2229-7-44 (PMC2000876; doi:10.1186/1471-2229-7-44)
Supplement: Additional file 1 — Genome size measurements. A discussion of our genome size survey in 22 A. thaliana accessions. [file 1471-2229-7-44-S1.pdf]

Genome size measurements

To assay variation in genome size and identify accessions with nuclear genome size larger and smaller than the sequenced accession Columbia (Col-0), we measured the genome size of 22 accessions acquired from the ABRC in Columbus, Ohio and the laboratory of Magnus Nordborg at the University of Southern California, using flow cytometry as described in Methods. Nuclear DNA content in the 22 accessions ranged from 150 to 170 Mbp. Genome size values are given in GS_table.pdf and graphed in GS_figure.pdf.

Schmuths *et al.* (2004) measured the genome size of 19 diploid Arabidopsis accessions using flow cytometry and *Raphanus sativus* (the cultivated radish) as an internal size standard. These investigators' largest and smallest measured values differ by 9% of the mean of all measurements while ours span 12% of the mean of our measurements. Three accessions are shared between the two sets; Col-0 has the smallest genome size in their measurements and is the fifth smallest in ours. The other shared accessions, Ag-0 and Tsu-0, are within 1% of the mean measured genome size in each set.
