# Supplementary material for: Large-scale polymorphism of heterochromatic repeats in the DNA of Arabidopsis thaliana
Source: BMC Plant Biol. 2007 Aug 16;7:44. doi: 10.1186/1471-2229-7-44 (PMC2000876; doi:10.1186/1471-2229-7-44)
Supplement: Additional file 2 — Survey of genome size variation in A. thaliana determined by flow cytometry. A table of the genome size variation we measured in Arabidopsis. [file 1471-2229-7-44-S2.pdf]

GS_table. Survey of genome size variation in *A. thaliana* determined by flow cytometry.

| Average genome size (Mbp) | Size relative to Col-0 | Accession name | Identification code | Standard deviation | CV | No. of individuals measured |
|---|---|---|---|---|---|---|
| 149.3 | 0.95 | Ta-0 | CS1548 | 0.8 | 0.6 | 2 |
| 153.1 | 0.98 | Lip-0 | CS1336 | 0.2 | 0.1 | 2 |
| 154.6 | 0.99 | Br-0 | 9A Br-0 A | 2.4 | 1.5 | 2 |
| 155.2 | 0.99 | Sp-0 | CS1530 | 2.6 | 1.7 | 2 |
| 156.5 | 1.00 | Col-0 | 8F Col-0 A | 0.8 | 0.5 | 5 |
| 157.5 | 1.01 | GOT-7 | 6E GOT-7 B | 2.2 | 1.4 | 2 |
| 158.0 | 1.01 | GOT-22 | 6F GOT-22 A | 0.5 | 0.3 | 2 |
| 158.3 | 1.01 | Rsch-0 | CS1490 | 3.6 | 2.3 | 2 |
| 158.4 | 1.01 | Ag-0 | 9C Ag-0 A | 0.2 | 0.2 | 2 |
| 159.0 | 1.02 | Yo-0 | 8E Yo-0 A | | | 1 |
| 159.9 | 1.02 | Nc-1 | CS1388 | 3.6 | 2.2 | 2 |
| 160.0 | 1.02 | Is-0 | CS1240 | 3.0 | 1.9 | 4 |
| 160.5 | 1.03 | Ct-1 | CS6674 | 1.2 | 0.7 | 2 |
| 160.5 | 1.03 | Per-1 | CS1444 | 2.0 | 1.2 | 2 |
| 161.2 | 1.03 | RRS-10 | 1B RRS-10 A | 0.3 | 0.2 | 2 |
| 161.4 | 1.03 | Tsu-0 | CS1564 | 0.7 | 0.4 | 2 |
| 162.9 | 1.04 | Eden-1 | 2A Eden-1 C | | | 1 |
| 164.5 | 1.05 | TAMM-2 | 6A TAMM-2 A | 0.8 | 0.5 | 2 |
| 166.9 | 1.07 | Kondara | 11H Kondara A | 0.4 | 0.2 | 1 |
| 169.1 | 1.08 | Nok-3 | CS6810 | 3.7 | 2.2 | 2 |
| 169.7 | 1.08 | Wt-5 | CS6896 | | | 1 |
| 169.9 | 1.09 | Loh-0 | CS1350 | 3.4 | 2.0 | 2 |

Units are millions of base pairs (Mbp). Accession identification code is either the ABRC stock number (those starting with CS) or that given by the laboratory of Magnus Nordborg. CV: Coefficient of variation is the standard deviation presented as percent of the mean. Prepared samples of each individual were measured two to three times.
