# Supplementary material for: Large-scale polymorphism of heterochromatic repeats in the DNA of Arabidopsis thaliana
Source: BMC Plant Biol. 2007 Aug 16;7:44. doi: 10.1186/1471-2229-7-44 (PMC2000876; doi:10.1186/1471-2229-7-44)
Supplement: Additional file 3 — Flow cytometry-determined average genome size and standard deviation (Mbp) of 22 Arabidopsis accessions. A figure presenting the mean genome size we measured in 22 Arabidopsis accessions. [file 1471-2229-7-44-S3.pdf]

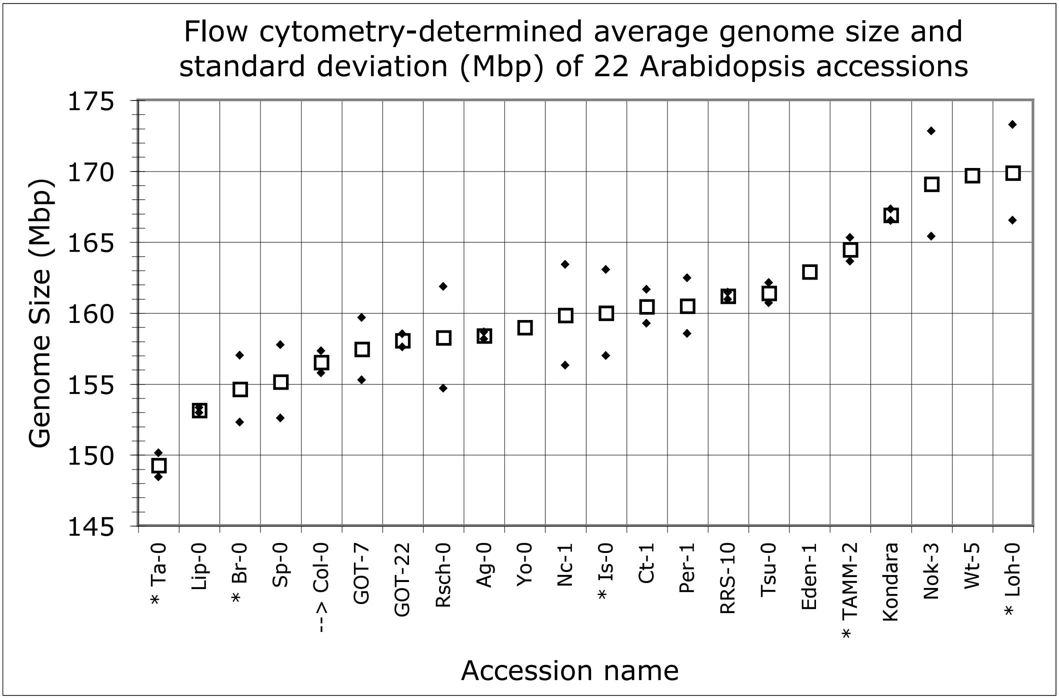

GS_figure. Mean genome size in millions of base pairs (Mbp) of 22 *Arabidopsis thaliana* accessions, measured using flow cytometry. Values are shown by a square marker; where more than one individual was measured the standard deviation of the separate means is given by diamond markers. Accessions selected for more detailed genome size measurement and assessment of repeat array size are m arked with asterisks; the sequenced accession Col-0 is noted with an arrow.
